# A tree-based conservation scoring method for short linear motifs in multiple alignments of protein sequences

**DOI:** 10.1186/1471-2105-9-229

**Published:** 2008-05-06

**Authors:** Claudia Chica, Alberto Labarga, Cathryn M Gould, Rodrigo López, Toby J Gibson

**Affiliations:** 1 EMBL Structural and Computational Biology Unit, Meyerhofstrasse 1, 69117 Heidelberg, Germany; 2 EBI European Bioinformatics Institute, Wellcome Trust Genome Campus, Hinxton, Cambridge, CB10 1SD, UK

## Abstract

**Background:**

The structure of many eukaryotic cell regulatory proteins is highly modular. They are assembled from globular domains, segments of natively disordered polypeptides and short linear motifs. The latter are involved in protein interactions and formation of regulatory complexes. The function of such proteins, which may be difficult to define, is the aggregate of the subfunctions of the modules. It is therefore desirable to efficiently predict linear motifs with some degree of accuracy, yet sequence database searches return results that are not significant.

**Results:**

We have developed a method for scoring the conservation of linear motif instances. It requires only primary sequence-derived information (e.g. multiple alignment and sequence tree) and takes into account the degenerate nature of linear motif patterns. On our benchmarking, the method accurately scores 86% of the known positive instances, while distinguishing them from random matches in 78% of the cases. The conservation score is implemented as a real time application designed to be integrated into other tools. It is currently accessible via a Web Service or through a graphical interface.

**Conclusion:**

The conservation score improves the prediction of linear motifs, by discarding those matches that are unlikely to be functional because they have not been conserved during the evolution of the protein sequences. It is especially useful for instances in non-structured regions of the proteins, where a domain masking filtering strategy is not applicable.

## Background

Linear motifs (LM) are short (3–10) amino acid sequences involved in numerous interactions including the modification-based regulation of protein function [1]. In particular, LM allow the formation of dynamic modular protein complexes due to the transient and low energy nature of the interactions they mediate [2]. Furthermore, LM are involved in targeting proteins to the appropriate cellular compartment [3]. Therefore, even if LM alone do not determine the complete molecular function of a protein, they give valuable information about the protein's role and/or position in the cellular function networks [4,5]. The experimental discovery of LM is time consuming and laborious, hence recently considerable research interest has focused on computational predictive approaches.

LM prediction is focused on the discovery either of new LM patterns, or the finding of new instances of already known patterns. ¿From the algorithmic point of view these two approaches represent different challenges. The identification of significantly over-represented sequence patterns in the former, and the distinction between true and false occurrences of a given pattern in the latter. The length of LM creates difficulties in both cases. The significance assessment of new patterns against the background probability distribution of LM is not straightforward due to their shortness. For the same reason, prediction of new LM instances by pattern matching is prone to produce a high proportion of false positives.

Methods for LM prediction take into account the biological context of those short sequences to evaluate the reliability of the newly predicted patterns or instances. Simple keyword association may sometimes be used to find significant connection between motifs and a specific function. That is the case for the EH1 motif, that occurs mainly in proteins containing domains with a transcription factor function [6]. The use of protein interactions has proven to be a fruitful approach to discover new LM patterns [7-11]. Currently DILIMOT [7], SLiMDisc [8] and more recently SLiMFinder [9] are the main tools for *de novo *LM discovery. The first one finds over-represented motifs in sets of proteins with a common functional attribute. The other two look for convergently evolved LM using evolutionary information derived from unrelated proteins that share a functional characteristic.

Resources for finding new instances of known motifs have begun to proliferate. Prosite is a large database of protein functional signatures. It initially included LM represented as regular expressions [12,13]. Currently, it is mainly devoted to domain profiles [14]. Scansite is a profile based search engine that predicts LM instances using the amino acid frequency information gathered from experimentally determined sites [15]. The ELM resource uses manually curated information about known eukaryotic LM to predict new instances, filtering out false positive matches with information about the structure, cellular compartment and species of the submitted sequence [16]. A similar approach has been implemented subsequently in other resources like the Minimotif Miner [17].

The use of evolutionary conservation has proved to be useful in the field of LM prediction. It improves the identification of truly functional instances of already known motifs [17-19] or allows to assess the strength of a new LM pattern [7,8]. The main assumption of this "phylogenetic footprinting" is that instances are conserved when they have a functional value and therefore conserved instances are less likely to be false positive occurrences of a motif.

When examining the conservation of LM, several specific problems arise. In contrast to domains that can be predicted using hidden Markov models [20], LM cannot be easily detected from a set of homologous sequences, since their conservation signal is not significant due to their length. That is why for motif prediction it is not enough to find a pattern inside a multiple alignment, but it is crucial to also consider the evolutionary relationships among the set of sequences. Moreover, LM tend to localise in structurally disordered segments of the proteins that are difficult to align [21]. This implies that the accuracy of the conservation scoring scheme also depends on the quality of the alignment.

An additional difficulty is the fact that LM have a non-linear pattern of conservation [22]. They are far more ephemeral than globular domains and their signature can appear or disappear as a result of single mutations. Ancestrality is not always necessary. This means that LM can appear *de novo *during protein sequence evolution, because they do not have to fulfil structural stability constraints in contrast to globular domains. LM losses are also possible in closely related sequences e.g. alternatively spliced forms.

Repetitive LM involved in the interaction with modular proteins, e.g. the *DPW *epsin motifs that mediate interaction with the adaptor protein AP-2, tend to be present in an inconstant number of copies in homologous proteins.

Finally, it is important to keep in mind that not all the amino acids forming a LM are equally informative. There are key positions, like the S/T/Y in a phosphorylation motif, that if changed result in the loss of function. Other positions accept more than one amino acid of the same physico-chemical group (e.g. acidic, hydrophobic), while some positions can be occupied by any amino acid. These differences have an impact in the definition of LM conservation.

This article presents a new scoring scheme that uses information about the degree of conservation to determine the reliability of a motif match or instance. The method has been developed inside the context of the ELM resource [16] in order to improve its predictive power without excessively degrading real time server performance for users. It is a three stage algorithm that efficiently manages to distinguish between true and randomly generated instances, keeping low both the false negative and false positive rates. A set of homologous sequences is defined. This set is used to reconstruct the evolution of the predicted instance in terms of the conservation of the corresponding regular expression. Subsequently, weights are assigned to the observed evolutionary events. The final conservation score (CS) is computed using all the gathered information.

## Results

In his work about scoring residue conservation, Valdar provides this advice: "Therefore, on the reasonable assumption that no method is perfect, a good conservation score should be no more complex than it needs to be so its deficits can be understood [23]."

**Figure 1:**
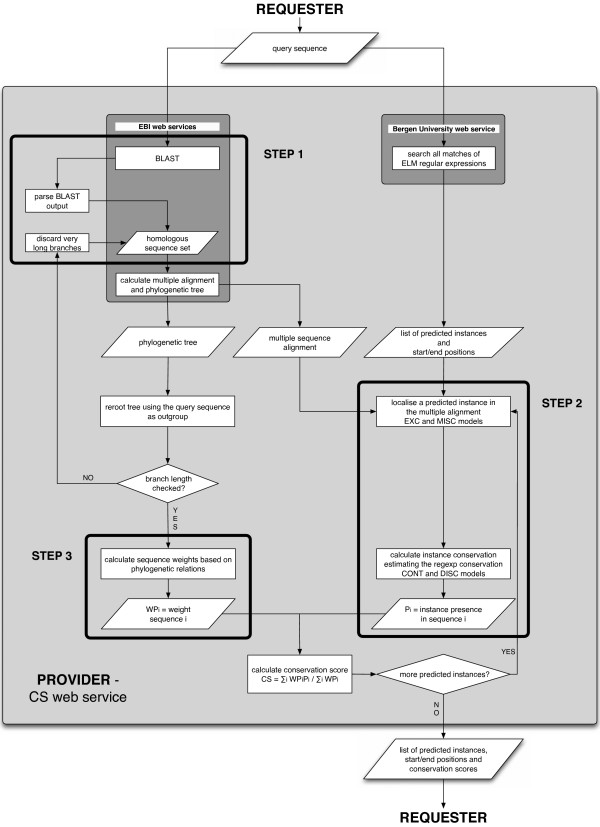
**Work flow of the conservation score implementation**. The conservation score is implemented as a Web Service (light grey) with two embedded Web Services (dark grey). It takes as input a protein sequence and gives as output the list of all the predicted instances with their positions and conservation scores. The Bergen University Web Service finds all the matches for ELM regular expressions in the query sequence. The EBI Web Service does the BLAST search, the multiple alignment and the phylogenetic tree calculation. The three main steps of the core algorithm are highlighted with black boxes.

The complete work flow of the CS is presented in Figure 1. It illustrates the Web Service implementation that takes as input a protein sequence and gives as output the list of all the predicted instances with their positions and CSs. The following sections describe in detail the ideas underlying each of the three main steps of the core algorithm.

### Step 1: Homologous sequence set definition

The set of homologous sequences is constructed by doing a BLAST search against the UniRef90 database [24]. The UniRef90 database is assembled by merging sequences and sub-sequences that are 90% or more identical, regardless of the source organism [25]. This is important to avoid redundant information inside the homologous sequence set. In fact, two very similar sequences can have the same motif at a certain position because they have not diverged enough in order to mutate those residues. In addition, a non-redundant database increases the speed of the BLAST search and improves the detection of distantly related sequences.

From the BLAST output, sequences that meet all of the following criteria are chosen: they are not annotated as hypothetical or predicted, they have 30% to 90% identity with the query sequence, and they are only 25% longer or shorter than the query sequence. This procedure aims to define a neighbourhood around the query sequence that is big enough to contain distantly related sequences. At the same time, it tries to avoid non-informative similar sequences like fragments or poorly predicted proteins that abound in the UniRef90 database.

This automatic parsing of the BLAST output gave good results for 70% of the sequences that contain known positive instances. The remaining 30% presented sequences that were very distant from the query sequence. These sequences affected the magnitudes of sequence weights, because they appeared as very long branches in the phylogenetic tree. They produced artificially small weight values when normalising by the total branch length (see Conservation score section). Moreover, these "orphan" sequences can diminish the alignment quality because they are only partially aligned, as previously demonstrated in [26].

**Figure 2:**
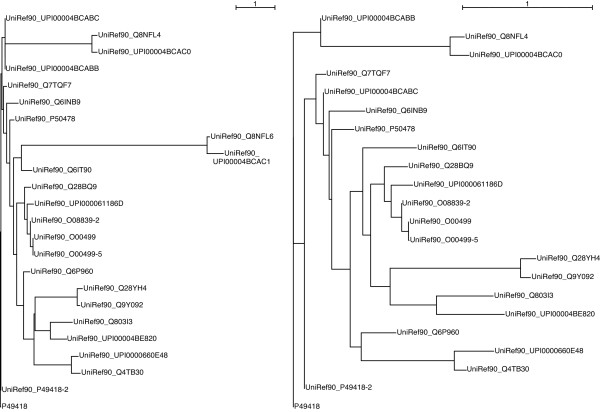
**Example of the impact of the branch length refinement**. After the automatic parsing of the BLAST output, outlier sequences that have a "non common" distance with the query sequence are discarded. The distribution of the branch lengths appears more even in the recalculated phylogenetic tree (right). This refinement step improves the alignment quality. It also prevents artificially small sequence weights being obtained upon normalisation by the total branch length. The trees are calculated using the neighbor-joining procedure in ClustalW; they are then rooted using the query sequence as outgroup, as explained in the Sequence weights determination section.

For these reasons a second stage of sequence quality check was introduced. In this refinement step the outlier sequences that have a "non-common" distance with the query sequence are discarded. Assuming that the phylogenetic distance between each homologous sequence and the query sequence follows a normal distribution, those sequences whose distance is in the right tail of such a distribution are eliminated. More precisely, those sequences whose distance is more than 2 standard deviations away from the average distance. An example of the change in the phylogenetic tree upon refining can be seen in Figure 2. As a result, the sensitivity of the scoring method, i.e. the ability to correctly score the known positive instances, improved by 16%.

### Step 2: Instance conservation determination

According to the scoring scheme, the predicted instance in the query sequence is considered to be conserved in an homologous sequence if the regular expression of the corresponding motif is located in the equivalent position given by the multiple alignment. Other matches to the regular expression that appear in the homologous sequences at different places are not considered evidence for conservation. This removes instances that could occur by convergent evolution. However, limitations in the sequence alignment quality may confound this requirement.

#### Motif sequence conservation

Consider the MOD_SUMO motif with regular expression [*VILMAFP*]*K.E *and the LIG_RGD motif with regular expression *RGD*. The former has 7 × 1 × 20 × 1 = 140 possible sequences that match the pattern, while the latter has only 1. More than 95% of the ELM motifs have degenerate regular expressions that represent more than one possible matching sequence, as in the MOD_SUMO case. This fact is relevant when quantifying the conservation of a predicted instance, i.e. the presence of the regular expression in a given homologous sequence.

One can consider the motif as a functional unit that requires the presence of all residues in the regular expression in order to be functional. The assumption underlying such a DISCONTINUOUS (DISC) model is that a mutation that disrupts the regular expression has the same cost, regardless of whether it happens in a stringent or a variable position. This corresponds to a deterministic model where the motif will be considered as conserved in the homologous sequence only if the regular expression is exactly matched. Only in this case will the presence value be 1. It is an appropriate approach for motifs such as phosphorylation sites, where the removal of a key residue will result in a non-functional instance. Despite this, it may be too rigorous for more "permissive" LM. For example, take the MOD_SUMO motif with regular expression [*VILMAFP*]*K.E*. Using the DISC model, both a mutation in the first position to an amino acid like *T *or a mutation in the second position to an amino acid different to *K *will give a presence value of 0. Nevertheless, *T *is physico-chemically similar to the set [*VILMAFP*]. Furthermore, the amino acid variability at the first position might be interpreted as a greater tolerance to mutations at that position. Indeed, a more "permissive" LM could still be functional even if the regular expression is not completely conserved, since they could allow for variations that are not considered by the regular expression. This could easily be the case, for example, for an instance in a yeast sequence of a motif that has been assigned from metazoan data.

Alternatively, the instance conservation can be calculated on a positional basis. This is the motivation for the CONTINUOUS (CONT) statistical model. It assumes that the motif's set of allowed sequences could be bigger depending on the proportion of variable and stringent positions in the regular expression. It weights the conservation of an amino acid at a stringent position higher than the conservation at a variable position. The rationale behind this is that the stringent positions are more informative. The information content per position is estimated using the Shannon entropy [27]. For an alphabet of 20 characters (i.e. 20 amino acids), the information content *I*_*i *_at a position *i *is:

$$I_{i}=\log_{2}(20)+\sum_{aa\in i}f_{aa,i}\log_{2}(f_{aa,i})$$

where *f*_*aa*,*i *_is the frequency of the amino acid *aa *at position *i *and the constant log_2_(20) is the normalisation value for an uniform distribution of amino acids in the sequence. In the particular case of a LM, the frequencies *f*_*aa*,*i *_can be defined from the regular expression. They are calculated assuming an uniform distribution of the amino acids allowed at each motif position. For the MOD_SUMO motif with regular expression [*V ILMAFP*]*K. E*: :$f_{aa,1}=\frac{1}{7}$, $f_{aa,2}=\frac{1}{1}$, $f_{aa,3}=\frac{1}{20}$ and $f_{aa,4}=\frac{1}{1}$. The fact that Σ_*aa*∈*i*_*f*_*aa*,*i *_= 1 implies that *I*_*i *_is a bounded value contained in the interval [0, log_2_(20)] = [0, 4.322], where higher values correspond to stringent positions that allow less amino acid variability and thus contain more information. The theoretical information content of a motif is defined as $I_{m}=\sum_{i=0}^{L}I_{i}$, where *L *is the motif length. The information content of the observed predicted instance *I*_*obs *_depends on the matching between the homologous aligned subsequence and the regular expression.

$$I_{obs}=\sum_{i=0}^{L}I_{i}\alpha_{i}$$

where α_*i *_= 1 if the observed amino acid *aa *is contained in the set of residues accepted for position *i*; otherwise α_*i *_= 0. The presence value in an homologous sequence *P*_*seq *_is the observed information *I*_*obs *_normalised by the theoretical motif information *I*_*m*_. It varies between 0 and 1, where 1 corresponds to an exact match of the regular expression and 0 indicates no match. Incomplete matches have presence values in between, depending on their similarity to the regular expression, i.e. the degree of motif conservation.

#### Motif position conservation

**Figure 3:**
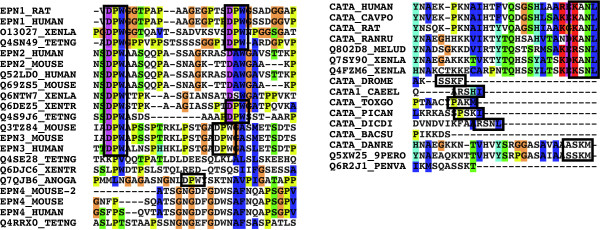
**Examples of motif misalignments in multiple sequence alignments calculated with ClustalW**. Certain linear motifs have characteristics that make them prone to be wrongly aligned by multiple sequence alignment algorithms. This is the case for repetitive motifs that occur in different number of copies per sequence, and often for those motifs located in the protein C-terminus. On the left, the repetitive instances of the *DP*[*FW*] epsin motif responsible for the binding with the AP2 adaptor complex. On the right, the C-terminus PTS1 motif involved in the targeting of the eukaryotic catalases to the peroxisome.

LM behave differently in multiple sequence alignments. In the best case, they appear as identifiable blocks of conserved residues. Sometimes, though, multiple sequence alignment algorithms fail to properly align them [28]. This is mainly due to the intrinsic difficulty of aligning disordered protein regions. Particular characteristics of some LM make this situation even worse. This is the case for repetitive motifs that occur in different numbers of copies per sequence, and often for those motifs located in the protein C-terminus (Figure 3).

The motif position conservation can therefore be approached in different ways. One possibility is to consider only the subsequence aligned onto the predicted instance. This implies the exact conservation of the instance position, therefore this model is called the EXACT (EXC) model. Another option is to search for the motif regular expression in a limited neighbourhood around the predicted instance. This MISALIGNMENT (MIS) model was tried by defining a window of 25 amino acids around the predicted match. It did not produce any significant improvement in the sensitivity of the method (see Testing section).

A real improvement of the sensitivity was achieved by using the EXC model and optimising instead the multiple alignment quality. Three fast multiple alignment algorithms were tested: ClustalW [29], Muscle [30] and Mafft [31]. The best results were obtained with Mafft, which improves the sensitivity of the model by 6% and 4% with respect to ClustalW and Muscle respectively.

More exhaustive multiple sequence alignment algorithms like ProbCons [32] which can perform even better than Mafft were not considered [28]. The reason for this is that the efficiency cost of using Probcons is not compatible with the real time implementation of the method.

### Step 3: Sequence weights determination

Once the instance conservation has been quantified in each homologous sequence, it is necessary to give a weight to each one of those presence values. As previously stated in [33] the quality of a match depends not only on the strength of the match (i.e. the conservation of the regular expression in this case), but also on the diversity of the sequences matched. Given the complexity of LM evolution, with both non-systematic losses or *de novo *occurrences, it is difficult to define a weighting scheme that is both specific and accurate. The general idea is that the conservation of the motif in distantly related sequences is more relevant than the conservation in closely related sequences. Conversely, the loss of the motif in distantly related sequences could be the result of the evolutionary divergence process (change of function) and is less informative than the absence of the motif in a close homologous sequence, especially if this is a recurrent fact.

Sequence weights can be calculated as a function of the divergence between the query sequence and each homologous sequence. This weight function is useful when the relative distance between sequences is approximately constant. When the distances among the homologous sequences are variable, which is often the case, a weight function based only on their divergence with the query sequence is not suitable. It can overweight presences in groups of sequences that are distant from the query, but very closely related among themselves. Therefore it becomes necessary to consider also the relative distances between all the homologous sequences. This can be done by taking into account the topology of the phylogenetic tree when assigning their weights.

Tree-based weights [34] have been used in protein profile searches to balance the sequence information contained in a multiple alignment. They are also used in ClustalW during the progressive alignment of the sequence subsets [29]. The weight assigned to each sequence is proportional to its distance from the root, and takes into account the number of neighbouring sequences. Given a multiple alignment of *N *sequences and the corresponding rooted phylogenetic tree, let *b*_1,*seq*_, ..., *b*_*i*,*seq*_, ..., *b*_*n*,*seq *_denote the path of *n *branches going from any sequence *seq *to the root, and *l*_*i*,*seq *_the length of a branch *b*_*i*,*seq*_. The Profileweight (*profw*_*seq*_) for *seq *is defined as:

$$profw_{seq}=\sum_{i=1}^{n}\frac{l_{i,seq}}{o_{i}}$$

where *o*_*i *_is the order of the node *i*, i.e. the number of leaves pending from that node. The phylogenetic tree is calculated using the neighbor-joining procedure in ClustalW [29]. Weights should be relative to the query sequence, therefore the tree has to be re-rooted using the query sequence as the outgroup. The weight for the sequence *seq *has to be corrected to consider the length of the final branch *L*_*f *_that leads to the root. The final weight for a sequence *seq *is

$$W_{seq}=profw_{seq}+\frac{L_{f}}{N-1}$$

where *N *- 1 is the order of the final node. The resulting weights are then proportional to the distance between the homologous and the query sequence and take into consideration the relative relationships among the homologous sequences. These weights are, of course, relative values that depend on the set of sequences and thus have to be standardised as shown in the next section.

### Conservation Score

The final score is calculated by adding the presence value *P*_*i *_of each informative sequence *i *weighted by the corresponding weight *W*_*i*_

$$CS=\frac{\sum_{i}W_{i}P_{i}}{\sum_{i}W_{i}}$$

where the normalisation constant in the denominator is the total weight for all the informative sequences in the multiple alignment. Normalisation is necessary to allow comparison of conservation scores coming from different homologous sequence sets. Moreover it produces a bounded score which varies between 0 and 1. The minimum value 0 means that the predicted instance is present only in the query sequence and the 1 indicates full conservation of the motif regular expression in all the informative sequences.

The non-informative sequences discarded from the summation are: those that diverge from the query sequence by more than *D*_*lim *_and have a presence value smaller than *P*_*lim*_; those with a gap bigger than 15 residues in the positions corresponding to the predicted instance. In other words, those sequences that could have lost the motif because of sequence divergence, or those sequences that do not have the motif because they are missing a larger subsequence e.g. alternatively spliced forms of the query sequence.

### Testing

To assess the accuracy of the models, their sensitivity and specificity was calculated in terms of the false negative and false positives rates (FNR and FPR). The known positive set is composed of 356 instances in the ELM database which are linked to experimental evidence and are coming from non-redundant sequences (see Additional file 1). From the published literature, it is not possible to gather the information required to define a suitable set of known negative instances. Therefore, a set of 1020 randomly chosen instances from intracellular protein sequences of the UniRef90 database was constructed (see Additional file 2). Those instances come from the same regular expressions and have an equal length distribution as the known positive instances. Moreover, they are located in protein regions with comparable amino acid distribution and conservation pattern to the true positive set. The procedure is the following:

1. Randomly choose one sequence *S *from the sequences annotated as intracellular or nuclear and longer than 100 residues in the UniRef90 database.

2. Using the IUPred algorithm [35] calculate the disorder regions *R*_1_, ..., *R*_*n *_in *S*. Motifs appear more frequently in unstructured regions of the proteins where the amino acid distribution and conservation is different from structured areas (globular domains). Therefore, in order to construct a set of random instances that is comparable in terms of background amino acid conservation with the known positive set, it is necessary to search for them only inside the *R*_*i *_protein fragments.

3. From the normal length distribution of known positives, randomly choose a length *L*. This is to avoid possible biases in the CS calculation caused by differences in the length distribution between the known positive and random instance sets.

4. Randomly choose a regular expression *regexp *of length *L*.

5. Search for a match to *regexp *in any of the *R*_*i *_subsequences longer that 10 residues.

The random instance set constructed is not a real negative set, but it gives an idea of the background noise that the CS models have to deal with. This set was tested to rule out the possibility of a bias in the amino acid composition that could affect the conservation of the random instances. For this purpose a different score that does not take into account any evolutionary information was used. (see Additional file 3, section A).

The results for sensitivity and specificity for a CS threshold of 0.58 are presented in Table 1. They were obtained assigning different values to the parameters *D*_*lim *_and *P*_*lim *_that determine the number of informative sequences for each instance. The TPRs and FPRs of the EXC CONT and EXC DIS models for all the different parameter combinations tried are shown in the Additional file 3, section B. Both EXC CONT and EXC DISC models achieve better specificity (1-FPR) and sensitivity (1-FNR) than the MIS DISC model. The latter seems unsuitable to the given problem since it scores 39% of the random instances as conserved and 16% of the experimentally confirmed instances as not conserved. Considering that sensitivity and specificity have contrasting tendencies, the best accuracy is achieved by the EXC DISC 1 and EXC CONT 1 models which minimise both FPR and FNR.

**Table 1:** Accuracy of the different models using different sets of parameters

Model	FPR	FNR	*D*_*lim*_	*P*_*lim*_
EXC DISC 1	0.22	0.14	0.30	1.00
EXC DISC 2	0.12	0.20	0.50	1.00
EXC CONT 1	0.19	0.17	0.50	0.80
EXC CONT 2	0.26	0.10	0.30	0.80
MIS DISC	0.39	0.16	0.30	1.00

The parameters *P*_*lim *_and *D*_*lim *_determine the number of informative sequences considered when calculating the conservation score of each instance (see Conservation Score section for further explanation). In all the cases, the false positive and false negative rates are calculated for the optimal threshold (0.58), which maximises both the model's sensitivity and specificity. *FPR *= 1 - *specificity *= $\frac{FP}{FP+TN}$*FNR *= 1 - *sensitivity *= $\frac{FN}{FN+TP}$

**Figure 4:**
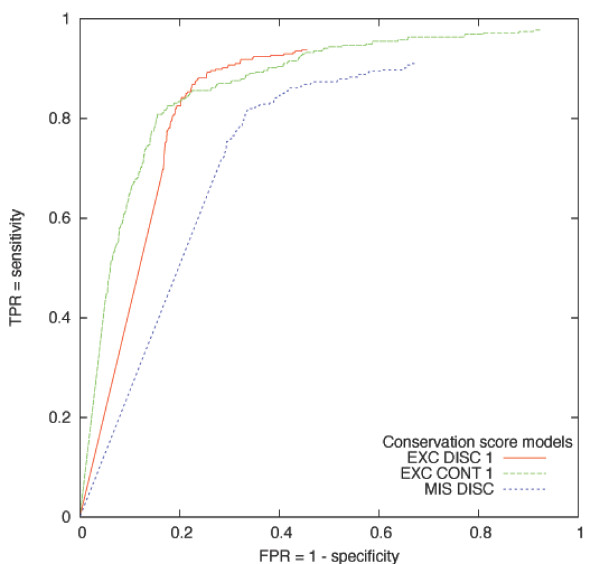
**ROC curves illustrating the performance of the different models**. Points in each ROC curve indicate the proportion of wrongly scored known negatives (false positive rate, FPR) versus the fraction of correctly scored known positives (true positive rate, TPR). Each point is calculated for a certain threshold inside the score range, [0, 1] for the conservation score. An ideal scoring method would be one arriving at the upper left corner, i.e. TPR = 1 and FPR = 0.

A comparison of the performance of these three models is presented in Figure 4. A ROC curve illustrates the model's performance in term of FPR and true positive rate (TPR = sensitivity). The further away the curve is from the diagonal (random score) and more towards the upper left corner (maximum sensitivity at no FPR cost), the better the model is. The ideal threshold corresponds to the point in the curve just before the start of the plateau where maximum sensitivity is reached at the lowest FPR. This threshold is 0.58 for all models. Independently from the chosen threshold, the EXC DISC cannot have a FPR higher than 0.45, but will always lose some of the known positives. On the other hand, the EXC CONT model can correctly score almost all the known positives but will increase considerably the FPR. The MIS DISC model is better than random, but shows the worst performance.

**Figure 5:**
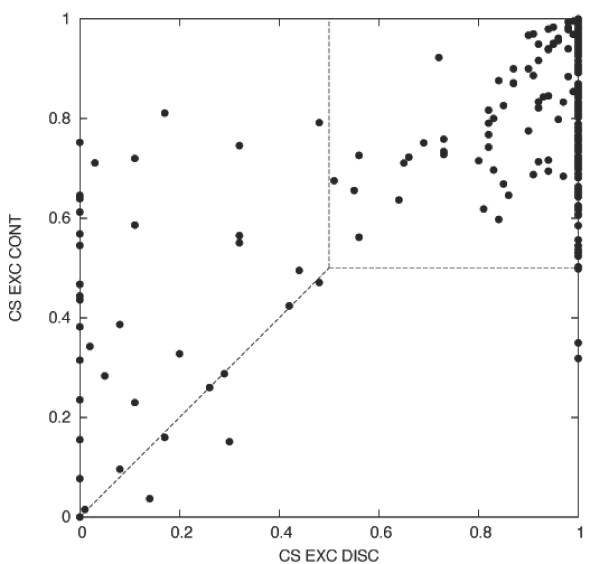
**Comparison of the EXC DIS and EXC CONT conservation scores for the known positive instances**. Each dot represents a known positive instance and its corresponding EXC DISC and EXC CONT conservation scores. Dots in the upper right square correspond to well conserved instances that score highly with both models. Dots on the left half and above the diagonal, indicate poorly conserved instances that are scored higher by the EXC CONT model.

When analysing the performance of the models using different sets of parameters, it becomes clear that the CONT and DISC models have different strengths. The EXC CONT model is good at scoring the known positive instances and can achieve the lowest FNR (0.10). This is because it gives higher scores than the EXC DISC model to the known positive instances that lack a very strong conservation pattern. Therefore it improves the chances of instances that show a partial conservation of the regular expression to be classified as conserved. This is illustrated in Figure 5 where the CSs calculated using both models are plotted for each known positive instance. Dots in the upper right square correspond to well conserved instances, that the two models can properly score. Dots on the left half and above the diagonal, indicate instances with lower conservation signal in the sequence alignment but still with a higher EXC CONT score. This property of the EXC CONT model is related to its capability of scoring the instances depending on the information content of the corresponding regular expression. Actually, the chi-square test for dependency between the CS and the average information content of the regular expression gives a significant result for the EXC CONT model (*P *= 0.02) but not for the EXC DISC (*P *= 0.13).

On the other hand, the EXC DISC model can reach the lowest FPR, meaning that it only scores 12% of random instances as conserved. This model manages to maximise the signal to noise ratio, that is to say it achieves the best separation between the score distributions of known positive and known negative instances. From an user point of view this means that for maximally stringent predictions the EXC DISC might be preferable. When the main interest is to identify a larger amount of conserved motifs, while tolerating some wrong predictions, then the EXC CONT model might be better.

### Implementation

The CS method has been implemented as a SOAP Web Service that enables both interactive (over the Web) as well as programmatic and thus systematic access to the scoring method. The CS Web Service is accessible at [36]. At this location the user can find a detailed description of the Web Service operations and a client implementation. The latter provides an example of programmatic access to the CS Web Service which would facilitate any remote tool integration.

The complete analytical workflow is illustrated in Figure 1. It comprises distributed Web Services that have been developed within and outside the ELM context. The starting point of the workflow is the ELMMatcher Web Service at the Bergen Center for Computational Science [37]. It accepts as input a protein sequence and returns a list of linear motif matches found within it. At the same time, the query sequence is compared against the UniRef90 sequence database using the WU-Blast Web Service at the EBI [38,39]. Results from this WU-Blast run are parsed and analysed as described in the Results section. The set of homologous sequences obtained is then used to generate a multiple sequence alignment using the MAFFT Web Service at the EBI [38,40]. This alignment is then re-used by the EBI's ClustalW Web Service to construct a phylogenetic tree using the neighbor-joining procedure in ClustalW [38,41]. Finally, all this information is used to calculate the CS and the results are returned to the user.

The CS Web Service should also be accessible via a graphical interface using the Cinema software. Cinema is a tool for annotated multiple alignment visualisation that is being developed in the context of the UTOPIA Toolset [42,43]. Instructions for using UTOPIA-Cinema as a front end for the CS Web Service are available at [44].

## Discussion

The present article presents an heuristic method for quantifying the conservation of a LM instance. The resulting score indicates the likelihood that the predicted instance is functional. It favours the conservation especially in divergent sequences, while tolerating some losses that might have happened during the protein sequence evolution. The scoring method can efficiently estimate the conservation of predicted instances in any sequence because it identifies common trends in LM evolution. It is not a method to predict the particular evolutionary history of each instance, which is a different and non-trivial task.

Conservation scores have been repeatedly implemented in order to improve LM prediction. The QuasiMotiFinder [18] algorithm uses a maximum likelihood-based model [45] to estimate the conservation of instances that resemble Prosite signatures. While being a very robust statistical approach, it is very time consuming for a real time application. The web-based tool Minimotif Miner calculates an evolutionary conservation score that requires the orthologues which are available for the completely sequenced eukaryotic genomes [17]. The significance of this score has been tested against a database of instances with randomised patterns. This could make it less suitable for distinguishing non-functional instances that match the same regular expression of the functional ones (e.g. specificity).

Recently, a conservation score for ranking predicted motif instances has been proposed [19]. This method follows a similar logic to the CS, in particular to the EXC CONT model. On our benchmark sets, it has similar selectivity but lower specificity than the CS EXC CONT model. Differences in the homologous sequence set definition might be the cause for the latter (for a detailed comparison between the two models see Addition file 3, section C).

The limits of the CS presented here can be better understood by having a closer look at the main causes for false negative (FN) and false positive (FP) predictions. Nearly half of the FN have a poor signal conservation that is not even recovered by the CONT model. Those instances are conserved only in one or two homologous sequences, or only in the closely related ones. Half of the remaining FN correspond to apparently *de novo *motifs that are present only in the query sequence and are therefore by definition not conserved.

Most of the observed FP correspond to random matches of LM regular expressions inside larger blocks of sequence conservation. Those instances also appear conserved in distantly related sequences. Therefore, it is likely that the individual amino acids are functional, either as part of a larger module or as part of an overlapping set of motifs It would mean that some of the FP might actually be TP. Nevertheless, it is difficult to distinguish whether those matches are true functional motifs or not using only their conservation information. This fact indicates that, for a more detailed study of LM evolution, the local distribution of amino acids around the predicted instance is an important element to take into account.

For the sake of generality and robustness it is necessary to check the principal dependencies of the method. As repeatedly shown in the Results section, the multiple alignment quality is crucial for the whole procedure. This implies a dependency on the total amount of information contained in the multiple sequence alignment. One way to estimate the total information in a set of homologous sequences is to calculate its total divergence, which is the sum of all the branches that separate each sequence from the root. The total divergence of the homologous sets of the sequences with known positive instances was calculated. A very small correlation was found between the total divergence and the CS. The Pearson correlation coefficient for the EXC CONT model is 0.14 and it is 0.07 for the EXC DISC model. This means that these models detect the conservation of the LM almost independently from the full sequence conservation pattern.

From the biological point of view, it is perhaps more interesting to investigate the number of different species that have to keep an instance in order to score it as conserved. On average, the known positive instances with CS equal to or higher than 0.58 are maintained in eight different species. For 66% of those instances, all species are vertebrates; for 22%, the different species also include invertebrates or even plants or fungi.

A more technical detail that still deserves some discussion concerns the suitability of regular expressions as the pattern matching tool for LM, compared with the profile/hidden Markov models used in resources like Scansite [46]. The fact that the CONT model achieves the lowest FNR would indicate that approaches that take into account the frequency of amino acids per position would be well suited to LM discovery. It is possible that the motifs in the known positive set tend to evolve by gradual sequence divergence before losing their functionality, instead of suddenly disappearing by point mutation. Since both behaviours have been observed in different motifs [22] and the results here presented are far from being comprehensive it is not possible to make a general statement about the evolutionary dynamics of LM. Nevertheless considering both FPR and FNR together, CONT and DISC models have similar accuracy for certain sets of parameters (EXC DISC 1 and EXC CONT 1). This indicates that, for the required level of resolution, regular expressions are appropriate for assessing the conservation of LM.

In a first application of the CS method, it has been used in validating detection of novel KEN box motifs [47]. The CS was significantly higher for KEN box motifs in cell cycle proteins, as compared to similar control motifs.

## Conclusion

Linear motifs are important modules in defining protein function. The conservation score method improves linear motif prediction especially in non-structured regions of the protein sequences, where the domain masking strategy for discarding non-functional instances is not applicable. The models developed are able to trace the conservation signal of differentially conserved true instances (false negative rates between 0.14 and 0.17). The divergence among sequences in the set is used to weight the conservation but the whole conservation score is independent from the total divergence inside the homologous sequence set. Moreover, the models have a high signal to noise ratio and therefore the false positive rates are low (0.19–0.22).

The conservation score is currently available as a Web Service at [36]. A graphical interface of the Web Service is provided by the UTOPIA toolset [43]. In the near future the conservation score will be added to the ELM resource.

## Abbreviations

LM: linear motif; CS: conservation score; EXC: exact; MIS: misalignment; CONT: continuous; DISC:  discontinuous; FPR: false positive rate; FNR:  false negative rate; TPR: true positive rate; FP: false positive; FN: false negative; TP: true positive; TN: true negative; regexp: regular expression.

## Authors' contributions

CC designed, developed and benchmarked. AL implemented the embedded EBI Web Services of the CS. CMG implemented a Java wrapper solution to encapsulate CC's python pipeline and expose the CS as a Web Service. RL hosts the EBI resources. TJG oversees ELM resource development.

## Supplementary Material

Additional file 1 Set of known positives. This set is composed of 356 instances in the ELM database which are linked to experimental evidence and are coming from non-redundant sequences. Click here for file

Additional file 2 Set of random instances. This set contains 1020 randomly chosen instances from intracellular protein sequences of the UniRef90 database. Click here for file

Additional file 3 Supplementary information. Find here more information about the test of bias in the random set, the optimisation of the model parameters and the comparison with Dinkel and Sticht method [19]. Click here for file
