# Supplementary material for: A tree-based conservation scoring method for short linear motifs in multiple alignments of protein sequences
Source: BMC Bioinformatics. 2008 May 6;9:229. doi: 10.1186/1471-2105-9-229 (PMC2396637; doi:10.1186/1471-2105-9-229)
Supplement: Additional file 3 — Supplementary information. Find here more information about the test of bias in the random set, the optimisation of the model parameters and the comparison with Dinkel and Sticht method [19]. [file 1471-2105-9-229-S3.pdf]

Supplementary Information

A Test of bias in the random set

The randomly chosen instances could be a biased set and this would affect the suitability to compare the evolutionary trajectory of the known positives. In order to check for possible bias in the amino acid composition that could affect the conservation of the random instances, we define the IUPred score, a different score that does not take into account any evolutionary information. This simple score (IUPred score) indicates the tendency of an instance to be in an unstructured region of the protein. We used the IUPred algorithm [1] and compute the average of the IUPred value for the amino acids belonging to the instance.

The IUPred score was calculated for all the randomly chosen instances and the 182 known positive instances located in unstructured regions of proteins (IUPs). The plot of the TPR

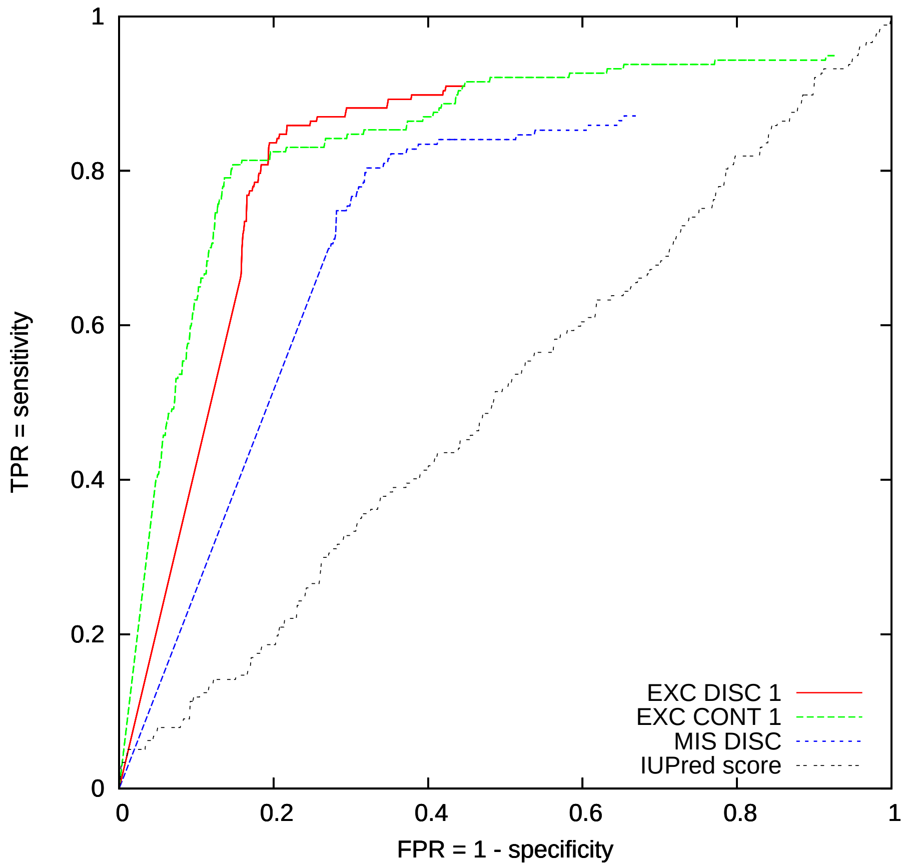

Figure S1. ROC curves of IUPred and CS scores. Points in each ROC curve indicate the proportion of wrongly scored known negatives (false positive rate, FPR) versus the fraction of correctly scored known positives (true positive rate, TPR). Each point is calculated for a certain threshold inside the score range, $[0, 1]$ for the CS (all models) and the IUPred score. An ideal scoring method would be one arriving at the upper left corner, ie. TPR=1 and FPR=0. The diagonal indicates a random scoring scheme.

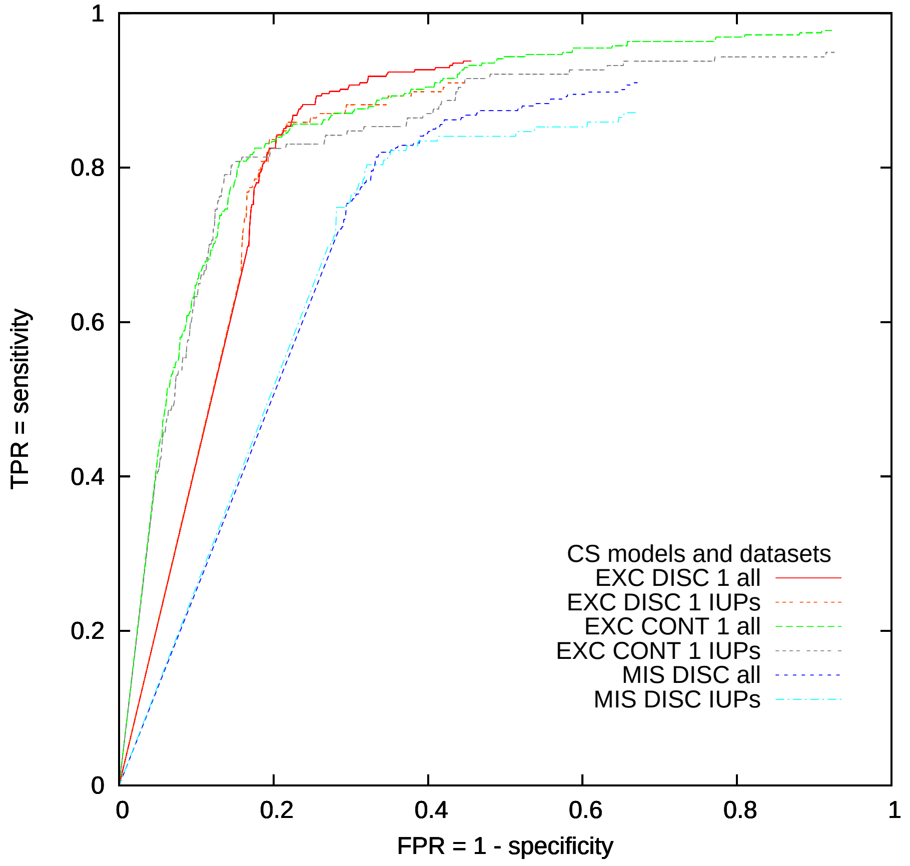

Figure S2. ROC curves of CS score using all known positives and those found in IUPs.

against the FPR for the IUPred score is a diagonal that corresponds to a uniform distribution of the score for the instances in the two sets (known positives in IUPs and randomly chosen instances). Moreover this indicates that the set of putative negatives is non-biased (Figure S1).

Given the fact that not all the 356 known positives could be used for the test described above, we compared the behaviour of the ROC curves calculated with both known positive sets: all instances and instances found only in intrinsically unstructured regions of proteins (IUPs) (Figure S2). For all three models the two curves differ only in the plateau region, after the ideal threshold where maximum sensitivity is reached at the lowest FPR. Indeed, there is no statistically significant difference between the CS distribution of both sets. The P-values of the Kolmogorov-Smirnov test range from 0.11 to 0.77 depending on the model used to calculate the CS.

B Optimisation of model parameters

model	TP	TN	FP	FN	FPR	FNR	D_{lim}	P_{lim}
EXC CONT	301	761	252	54	0.25	0.15	0.3	0.3
	315	686	327	40	0.32	0.11	0.3	0.4
	322	650	363	33	0.36	0.09	0.3	0.5
	322	677	336	33	0.33	0.09	0.3	0.6
	320	724	289	35	0.29	0.10	0.3	0.7
	318	750	263	37	0.26	0.10	0.3	0.8
	316	750	263	39	0.26	0.11	0.3	0.9
	294	786	227	61	0.22	0.17	0.4	0.3
	307	727	286	48	0.28	0.14	0.4	0.4
	312	697	316	43	0.31	0.12	0.4	0.5
	313	712	301	42	0.30	0.12	0.4	0.6
	312	752	261	43	0.26	0.12	0.4	0.7
	311	775	238	44	0.23	0.12	0.4	0.8
	311	785	228	44	0.23	0.12	0.4	0.9
	283	830	183	72	0.18	0.20	0.5	0.3
	291	792	221	64	0.22	0.18	0.5	0.4
	295	769	244	60	0.24	0.17	0.5	0.5
	298	781	232	57	0.23	0.16	0.5	0.6
	297	803	210	58	0.21	0.16	0.5	0.7
	295	820	193	60	0.19	0.17	0.5	0.8
	293	825	188	62	0.19	0.17	0.5	0.9
EXC DISC	304	787	226	51	0.22	0.14	0.3	1.0
	283	840	173	72	0.17	0.20	0.4	1.0
	251	892	121	104	0.12	0.29	0.5	1.0

Table S1. Performance of the EXC CONT and EXC DIS models for different values of the D_{lim} and P_{lim} parameters. The number of true/false positives/negatives and their corresponding false positive and false negative rates are shown for the significant combinations of the two parameters. The parameters P_{lim} and D_{lim} determine the number of informative sequences considered when calculating the conservation score of each instance (see Conservation Score section for further explanation). For the EXC DISC model only the D_{lim} is considered, since the only possible value for P_{lim} is 1.

C Comparison with Dinkel and Sticht score

Recently, another conservation score for ranking predicted motif instances has been proposed [2]. This method retrieves homologous sequences by a BLAST search and sorts them according to their similarity with the query sequence. Then it looks for the presence of the predicted instance in the pairwise alignments with the homologous sequences. The method takes into account the variability of the motif pattern when assessing the presence of an instance in the homologous sequence. Each presence is weighted depending on the sequence similarity between the homologous sequence and the query. A conservation score is then calculated averaging the weighted presences for a number of homologous sequences (average conservation score, ACS). The final conservation score for the given instance is the maximum average conservation score (MCS) obtained using different numbers of homologues.

In general the method of Dinkel and Sticht follows a similar logic to the CS, in particular to the EXC CONT model. The main differences with the CS method are: the absence of a "closed" homologous sequence set, in the sense that the number of considered homologues depends on the maximisation of the ACS; the use of pair wise alignments instead of multiple sequence alignments.

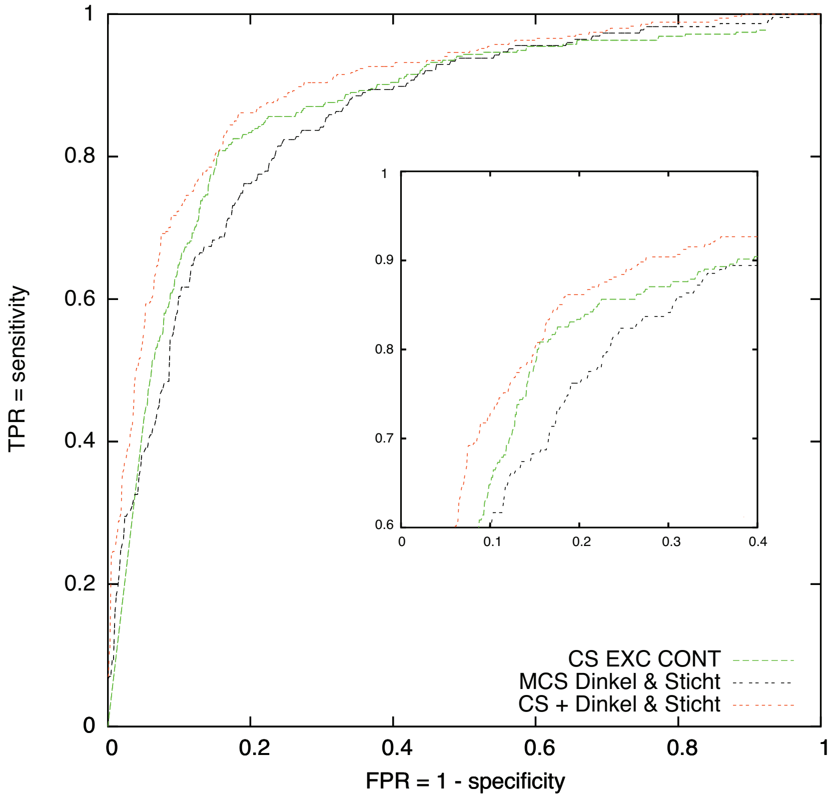

Figure S3. ROC curves comparing the performance of the MCS (Dinkel & Sticht) and the EXC CONT model of the CS. Both methods are equally suitable for recovering known positive instances, e.g. they have the same selectivity. In the low FPR region, the EXC CONT model shows better specificity than the MCS. The best performance is achieved when combining both models (CS + Dinkel & Sticht)

In spite of the general resemblance between the two methods, Dinkel and Sticht report a recovery rate (e.g. sensitivity) of 75% for their method [2]. This result differs from the 0.83 sensitivity found for the CS EXC CONT model. The most natural explanation for this would be the difference between our known positives set and theirs. Dinkel and Sticht used all the 675 ELM instances (567 after filtering for sequence redundancy). For the CS benchmark, instead, only the 356 ELM instances linked to experimental evidence were used. In order to test this hypothesis, we implemented their method and calculate the MCS of our known positive and known negative instances.

The resulting ROC curves show that the MCS and the CS EXC CONT model have similar sensitivity (Figure S3). Indeed both methods take into account the degenerate nature of linear motifs patterns. This seems to be important when scoring instances that are known to be functional (for further discussion see Testing section and Figure 5, in main article).

Nevertheless, there is a difference between the two methods in the high TPR and low FPR region of the ROC curve (see inset Figure S3). There, the CS EXC CONT model reaches the same TPR as the MCS at lower FPR cost. The difference in FPR ranges from 0.05 to 0.10. It is possible that the two main differences between the methods explained above are responsible for the overscoring of some of the randomly chosen instances. The maximisation of the ACS might not be ideal in a prediction framework where it is necessary to distinguish among functional and non functional instances. It could "overscore" random matches. Moreover, the use of pairwise alignments could increase the probability of finding the random instances in the homologous sequences and therefore of scoring it as conserved. This is less likely to happen in a multiple sequence alignment.

For a final test, we combined both methods. We constructed the set of homologous sequences according to our approach (for a detailed description see section Step1: Homologous sequence set definition, in main article). Using the corresponding multiple sequence alignments, we calculate Dinkel and Sticht ACS averaging the weighted presences for sequences in the homologues set. The best performace is achieved when the strong points of both methods are put together (red line in Figure S3).

References

[1] Dosztányi Z, Csizmok V, Tompa P, Simon I: **IUPred: web server for the prediction of intrinsically unstructured regions of proteins based on estimated energy content**. *Bioinformatics* 2005, **21(16)**:3433–4.

[2] Dinkel H, Sticht H: **A computational strategy for the prediction of functional linear peptide motifs in proteins**. *Bioinformatics* 2007, **23(24)**:3297–303.
